# Impact of Income Inequality on Urban Air Quality: A Game Theoretical and Empirical Study in China

**DOI:** 10.3390/ijerph18168546

**Published:** 2021-08-13

**Authors:** Feng Wang, Jian Yang, Joshua Shackman, Xin Liu

**Affiliations:** 1 Department of Applied Economics, School of Economics and Business Administration, Chongqing University, Chongqing 400044, China; wangfeng2008@cqu.edu.cn (F.W.); yangjian9109@163.com (J.Y.); 2 International Business and Logistics Faculty, California State University Maritime Academy, Vallejo, CA 945900, USA; JShackman@csum.edu

**Keywords:** income inequality, air pollution, environmental awareness, mixed-strategy games

## Abstract

Income inequality and environmental pollution are of great concern in China. It is important to better understand whether the narrowing of income inequality and environmental improvement contradict each other. The study aims to investigate the linkage between income inequality and environmental pollution. To illustrate the interplay between different income groups on environmental issues, we apply a mixed-strategy game. Based on the game-theoretic analytical result, the probability of residents supporting clean energy and environmental protection decreases as income inequality widens and increases as inequality narrows. This empirical study is based on the proportion of coal consumption and urban air pollution data from 113 key environmental protection cities and regions in China. The air quality data are from the National Environmental Air Quality Monitoring Network published in the China Statistical Yearbook from 2014–2018. Convincing results show that regions with higher income inequality suffer severe smog and related pollution and that economies with narrow income disparity experience significant improvements in smog and pollution control, with the expansion of the proportion of clean energy use. The results also provide no evidence of the impact of per capita income on pollution. We studied the relationship between individuals of different wealth levels within an economy, within a repeated-game setting. The finding suggests that the distribution of growth impacts pollution. Imposing higher taxes on air polluters while transferring the revenue to the lower-income group is suggested.

## 1. Introduction

In addition to the debate over the balance between economic growth and environmental protection, scholars have begun to discuss the role of economic inequality, which measures how unevenly income is distributed throughout a society. Few countries other than China illustrate the need for more research on the relationship between inequality and the environment. China has experienced rapid economic growth over the last two decades [1] that has caused crisis levels of inequality and pollution. For example, China’s Gini coefficient (a measure of the gap between the rich and poor) was recently estimated as 0.47 (http://www.stats.gov.cn/ztjc/zdtjgz/yblh/zysj/201710/t20171010_1540710.html, 10 October 2017), one of the highest levels of inequality in Asia, where the average level of the Gini coefficient was estimated as 0.36 (https://data.adb.org/dataset/gini-coefficient-asia-and-pacific, 8 November 2016). [2]. This number is approaching a “danger line” of 0.5 [3] associated with widespread social instability and stagnation of development [3,4,5]. In addition, air pollution is at levels that harm human health in most of China’s cities. Of the 338 cities monitored by China’s Ministry of Environmental Protection in 2015, 73 cities fulfilled national standards for air quality [6], which refers to the air pollution level in these cities. In this study, we mainly focus on the linkage between income inequality and urban air quality in China. In other words, we want to clarify how the increasing income disparity potentially influences air pollution. Under the realization of that common prosperity and environmental governance are important issues in the long-term plan of the Chinese government, it is crucial for policy-makers to clarify the relationship between income inequality and environmental pollution, which is still academically controversial.

The results of the theoretical analyses, specifically within the game-theoretic model, suggest that inequality is associated with greater environmental pollution, which is empirically confirmed through the use of data from 113 Chinese cities for most forms of air pollution used within this study. These data depict the current status of China as having a large degree of inequality among individuals and cities. Multiple types of air pollution are employed as measures of environmental damage. Air pollution, most likely influenced by income inequality [7], has become a major factor threatening the health of residents. According to the World Health Organization [8], 1,150,296 Chinese citizens have died because of air pollution, and ceasing this trend is one of the most urgent environmental issues for policymakers and citizens.

The empirical results show that income inequality aggravates air pollution, measured by annual average fine particulate matter (PM2.5) concentration, and further prove that in addition to PM2.5, income inequality is positively correlated with other air pollution indicators. The results also show no evidence of the impact of per capita income on pollution. For endogeneity concerns, we use the urbanization rate as the instrument variable (IV). Based on the two-stage least squared (TSLS) regression analysis, we confirm the robustness of the empirical result. This finding suggests that the distribution of the growth, as opposed to economic growth, impacts pollution. From the perspective of policy-making, imposing higher taxes on air polluters while transferring the revenue to the lower-income group is suggested.

## 2. Literature Review and Development of Hypotheses

### 2.1. Trade-Off between Economic Growth and the Environment

Studies have argued that at the initial stage of economic development, large amounts of natural resources are consumed, leading to the discharge of waste and build-up of pollutants, this ultimately compromises social well-being [9]. However, in the long run, economic growth leads to improved environmental protection. Such viewpoints have been well summarized and empirically supported by prior research e.g., [10,11]. One of the most influential theories is the Environmental Kuznets Curve (EKC) [12] derived from the well-known Kuznets Curve [13,14]. According to the curve, environmental damage is positively correlated with economic development in pre-industrial economies, but in post-industrial economies, environmental damage declines with economic development. However, subsequent studies have challenged the aforementioned findings or deeply examined the impact of income and on the environment [15,16,17]. Subsequently, economic experiments and other new methods have been used to validate this issue e.g., [18]. Thus, because some environmental factors vary with a change in residents’ income, considering potential associations between income inequality and environmental protection is valuable and reasonable.

### 2.2. Empirical Evidence on the Linkage between Inequality and Emissions

Much of the empirical literature on inequality and emissions has obtained contradictory or inconclusive results. Research on income inequality and the environment has focused on carbon emissions. The positive association between inequality and carbon dioxide (CO_2_) emissions has been examined at the country level [19,20] and state level (provincial level) [4,21,22]. However, contradictory results indicate low [23,24] or negative [25] correlation between inequality and CO_2_ emissions.

Scholars have begun investigating air pollution and proposed smog prevention and control recommendations [26,27]. Research on non-CO_2_ emissions and inequality has shown similarly mixed results and has examined forms of air pollution such as sulfur dioxide (SO_2_) or air particulates [28,29,30,31,32]. A majority of the literature argues that income inequality positively impacts emissions [33,34]. At the national level, some scholars believe the turning point of EKC will be realized at a lower economic level with a narrower income gap [34]. However, the effect of such a relation in different countries may vary according to the level of development [33]. Controversial points on this issue mainly distinguish on whether the types of pollutants and sample differences are the key determinants in influencing the relationship between inequality and pollution [35,36].

Additionally, two studies have examined the relationship between inequality and overall indices of environmental pollution that include air pollution as part of their weighted index [37,38]. Interestingly, some research argues that air pollution can also influence income inequality. For example, pollution may have different impacts on individual activities depending on the time dimension and eventually exacerbate the income inequality [39]. Besides, income inequality may also affect the impact of environmental pollution on different groups and make some more vulnerable [40]. These findings pose a potential endogenous problem when modelling income inequality and air pollution.

Because the relationship between inequality and environmental pollution remains unaccounted for, additional discussions are necessary. This study extends CO_2_-oriented research [4,21,24] by examining the relationship between economic inequality and multiple measures of daily air quality beyond CO_2_. We also extend the research on provincial-level data [37,38] by using city-level data and examining the impact of inequality on multiple and specific measures of air quality.

### 2.3. Game Theoretical Approach and Other Formal Models

Informed by the research on the relationship between economic activities and climate or environment change [41,42,43], and based on non-cooperative games [44], we created a normative non-cooperative game and its Nash equilibrium. The non-cooperative game between “rich” and “poor” economies demonstrates a typical prisoner’s dilemma. If the strategic space is defined as a limited continuously derivable set, a social dilemma of sub-optimal Nash equilibrium is observed [45].

For dynamic games with perfect information, backward induction can be used to obtain the subgame perfect Nash equilibrium, which entails that different players tend to reach consensus over time under dynamic conditions [42,46,47]. The endlessly repeated prisoner’s dilemma can provide a more realistic depiction, and the literature has concluded that there are many subgame perfect Nash equilibriums under trigger conditions [48,49]. Studies have found that different governance measures, including subsidies, taxes, and other incentives, could be used to change the game equilibrium and ultimately enhance the efficiency of the sub-optimal equilibrium [50]. Further research investigated the game of environmental protection between China and Japan by examining acid rain and suggested that Japan should subsidize China in the area of acid rain control [51]. This illustrates the difficulty of achieving cooperation without a central government to resolve conflicts through the use of subsidies or regulations.

The academic community has also endeavored to examine cooperative games of emission reduction. Optimal emission reduction was considered possible under extensive cooperation conditions [52]. Cooperation had positive externalities, and even small-scale cooperation could lead to emission reduction [53]. Some scholars have used implementation theory to study the game of pollution control [54] and found that limited cooperation in dynamic games could result in more efficient pollution control than simply being a endeavored free rider.

In addition to the game-theoretic approach, other formal models are applied. Other factors, for example, type of democracy [55] and social power [28,56], have often been included in the analytical frame of income inequality and environment. In some cases, the importance of concavity in the relationship between income and environmental damage is emphasized [30]. However, the existence of concavity or convexity remains controversial [31,57].

Most of these models have made strong assumptions regarding how much power the wealthy have, strong preferences for pollution by the wealthy, what type of government is best, or household consumption patterns. The model presented in this study is designed to have minimal assumptions (other than standard assumptions about preferences for income and environmental protection). We draw similar conclusions on the impact of inequality on pollution but use fewer assumptions. We also use a game-theoretic approach to explore a greater number of outcomes that depend on the distribution of wealth.

As mentioned previously, the role of inequality between nations has been modelled in a game-theoretic approach [51,58,59] and has highlighted the difficulties of obtaining cooperation between developed and developing countries on mutually beneficial environmental policies. Although these studies illustrate the challenges of developing countries (e.g., China) cooperating with developed countries on issues such as climate change, they do not provide guidance on obtaining cooperation between individuals of different wealth levels within a country. This study extends the analysis of these studies by using a repeated-game approach to model the impact of inequality on environmental protection within a single country rather than among countries.

In summary, if EKC holds in China, then environmental pollution may further deteriorate with the expansion of the income gap before the turning point of the curve. Conversely, after reaching the turning point, the narrowing income disparity would result in environmental improvement. Therefore, based on the urban air pollution condition, we establish the following two hypotheses, which will be theoretically analyzed using a mixed-strategy game and empirically tested in the due course.

**Hypothesis** **1** **(H1).** *There is an inverted U-shaped relationship between economic growth and air pollution in urban China according to the EKC hypothesis.*

**Hypothesis** **2** **(H2).** *Expansion of income inequality aggravates air pollution in urban China.*

## 3. Materials and Methods

### 3.1. Game-Theoretic Model

This section selects an ongoing environmental protection project aiming to address environmental pollution and improve environmental quality, studies two groups, namely, the high-income group and low-income group, and analyses how well the groups accept the project through the use of mixed-strategy games. As two independent decision-makers, the two groups independently decide whether they accept the project and if they choose to make optimal self-benefiting decisions according to the utility maximization principle. As the macro-regulatory body, the government encourages and promotes residents to accept the project and facilitates project realization by providing subsidies and levying the tax. This proposed model analyses the two groups’ optimal decision-making under the two different approaches.

We suppose that residents in an economy comprise non-cooperative game players, with their income following a normal distribution according to which residents are defined as $i\in (\mu ,\sigma^{2})$, where *μ* denotes the mean of residents’ income and *σ* denotes its standard deviation, and every resident has an identifiable income characteristic $I_{i}\in R^{+}$. Income is an exogenous variable in this benchmark model. Without loss of generality, income is defined as $I_{i}\in (0,1]$. The government does not participate in the game at this stage.

When making the decision, residents independently choose to accept or reject in a non-cooperative manner. Their income follows a normal distribution with the median defined as $\bar{I}$. The high-income group is defined as those with higher-than-median income, and the low-income group is defined as those with lower-than-median income. Real numbers are defined as $P_{i}\in [0,1]$, and the accompanying probability of strategy is $P_{i},i=1,2,3\ldots n$, assuming the accompanying probability of residents choosing to “accept” is $P_{i}$ and that of residents choosing to “reject” is $1-P_{i}$. *P* is positively associated with income. When making the decision, the players should consider both their preference and common social factors; that is, residents’ preferences and common social factors which jointly shape their decisions.

The environment is usually considered as a sort of public goods. This study refers to some relevant research [60,61,62] on the consumer utility function setting in the supply of public goods. It is also combined with the classic assumption of income utility and makes the following assumptions about the utility function of the individual. We suppose that the utility is a discrete finite set. The utility is reduced to U(I,E) without loss of generality, where *E* denotes the environmental quality coefficient. We assume that the high-income individuals have high marginal utility because they can vote for the environmental project at a certain cost other than the loss of income. Low-income individuals may experience the loss of utility by paying a price for the environmental project that may be higher than the gain from an improved environment. The utility function satisfies the following conditions:

$$\begin{array}{l} I_{i}>I_{j}\Leftrightarrow U_{i}(I_{i},E^{*})>U_{j}(I_{j},E^{*})\\ E_{i}>E_{j}\Leftrightarrow U_{i}(I^{*},E_{i})>U_{j}(I^{*},E_{j}) \end{array}$$

where E* and I* denote any *E*-level and *I*-level, respectively. We assume that the efforts to improve the environment are because of individuals’ financial contributions deducted from their incomes. Each individual pays the same amount of donation to support a project that significantly increases the utility of the environment. The players can either approve or reject the project by paying or not paying. The players’ utilities are composed of income and environmental utility.

**Proposition** **1** . *In a mixed-strategy environmental protection game* $G=\left\{S_{1},\ldots S_{n};u_{1}\ldots u_{n}\right\}$ *for any strategy* $S_{i}=\left\{S_{i1}\ldots S_{ik}\right\},i\in \left\{1,2\ldots n\right\}$*, if there is no authoritative intervention by the government and residents choose the game independently, there is only one pure strategy Nash equilibrium,* θ=0,  γ=0. θ *is the accompanying probability of the high-income group choosing to “accept” and γ is the accompanying probability of the low-income group choosing to “accept”*.

In a typical Nash equilibrium, spontaneous environmental protection behaviors are unlikely. That phenomenon is called a prisoner’s dilemma. Therefore, it is necessary to introduce government intervention to solve the “uncovered market problem” [63]. When making the decision, individuals should consider their preferences and common social factors. We assume that there is a strictly monotonically increasing utility function of income. Hence, in an outcome of a non-cooperative game, rational individuals reject the project, inevitably leading to environmental damage and reductions in social benefits.

We suppose that there is a society-administering government that regulates the emission of pollutants through the use of tax. Now, we introduce subsidy $t_{i}$ into the game model as compensation for environmental impact. The subsidy is related to income: $t_{i}\in N^{+},i\in \left\{1,2,3\ldots n\right\}$.

**Proposition** **2** . *In a mixed-strategy environmental protection game* $G=\left\{S_{1},\ldots S_{n};u_{1}\ldots u_{n}\right\}$, *when the government provides subsidies for environmental protection,* $s_{i}\in N^{+},i\in \left\{1,2,3\ldots n\right\}$ *exists, satisfying the mixed-strategy Nash equilibrium* $S^{*}=\left\{S_{1}{}^{*},\ldots S_{n}{}^{*};u_{1}{}^{*}\ldots u_{n}{}^{*}\right\}$, and the accompanying probability for the high-income group and the low-income group to “accept” is $\theta^{*}$ *and* $\gamma^{*}$, respectively.

The subsidy of the environmental project does not increase the income level of high-income or low-income individuals. Rather, the income levels of both subsets decrease. However, some may have a higher utility in aggregate when environment utility is included in the function. Without the loss of generality, we suppose that the income of the high-income group and the low-income group are $\bar{I_{H}}$ and $\bar{I_{L}}$, respectively. To further understand the Nash equilibrium in the subsidy situation, we suppose that the tax on the high-income group and the low-income group are $\bar{t_{H}}$ and $\bar{t_{L}}$, respectively.

**Proposition** **3.** *The defined mixed-strategy environmental protection game*$G=\left\{S_{1},\ldots S_{n};u_{1}\ldots u_{n}\right\}$*has and only has one mixed strategy Nash equilibrium*$S^{*}=\left\{S_{1}{}^{*},\ldots S_{n}{}^{*};u_{1}{}^{*}\ldots u_{n}{}^{*}\right\}$and has accompanying probability$P_{i}\in [0,1]$.

We define this as: $\Delta I=\bar{I_{H}}-\bar{I_{L}}$.

**Proposition** **4.** *In the mixed-strategy Nash equilibrium* $S^{*}=\left\{S_{1}{}^{*},\ldots S_{n}{}^{*};u_{1}{}^{*}\ldots u_{n}{}^{*}\right\}$ *of the mixed-strategy environmental protection game* $G=\left\{S_{1},\ldots S_{n};u_{1}\ldots u_{n}\right\}$ *,* θ *and* ΔI *are monotonically negatively correlated, and* γ *and* ΔI *are monotonically negatively correlated.*

Proposition 4 indicates that in a mixed strategy game, the probability of an environmental protection policy being supported decreases as income inequality widens and increases as income inequality narrows. Notably, we assume that the income $I_{i}\in (0,1]$ is normally distributed; thus, the low-incomers have negative utilities change when there is public expenditure on investment, and high-incomers improve their utilities when there is public investment in the environment. If the income inequality is ΔI→0, the proportion of residents supporting an environmental protection policy reaches a maximal value. In a loose definition, the environment is more likely to maintain a high level when the income is less unevenly distributed than when it is more evenly distributed. This conclusion forms the most important normative research finding of this paper, from which we can deduce that residents’ willingness to adopt environmental protection measures is weak in locations where income inequality is widespread; therefore, achieving meaningful environmental improvement is difficult. In locations with moderate income inequality, residents’ willingness to adopt environmental protection measures is strong, thus, environmental protection can be achieved at a lower cost.

### 3.2. Model Specification

We attempt to empirically verify the findings from micro-level foundation theory research. The core variables of this empirical research are air quality and income inequality in Chinese cities. We study the income inequality of major cities and environmental coefficients in 2014–2018, thus attempting to identify the empirical relationship between them. We focus on cities because the greatest amount of air pollution is generated and spread within urban areas. However, because the geographic range of Chinese administrative cities’ may include rural areas, we also discuss the detailed urban–rural income ratios.

To examine the impact of income inequality on air quality, we constructed a regression model:

(1) $$AIR_{i,t}=\beta_{0}+\beta_{1}URI_{k,t}+\beta_{2}X_{i,t}+\mu_{i}+\varepsilon_{i,t}$$

where, subscripts *i*, *k,* and *t* denote cities, provinces, and years, respectively. $\mu_{i}$ controls for the individual fixed effect. $\varepsilon_{it}$ is a random error term. *AIR_i,t_* is the air quality of city *i* in year *t,* measured by seven indicators: the annual average SO_2_ concentration (μg/m^3^), annual average nitrogen dioxide (NO_2_) concentration (μg/m^3^), annual average inhalable particulate matter (PM10) concentration (μg/m^3^), the 95th percentile of daily average CO concentration (μg/m^3^), the 90th percentile of the daily maximum 8-h average O_3_ concentration (μg/m^3^), the average annual concentration of fine particulate matter (PM2.5) (μg/m^3^), and the number of days with air quality reaching or exceeding grade II (good air quality days [GAQD]) (according to the national standard “Air Quality Standard (GB 3095-2012)” issued by the Ministry of Ecological Environment of the People’s Republic of China in 2012, if the concentration of a certain type of air pollutant in a city exceeds the concentration limit of air quality grade II, it is determined that the air quality of that day does not reach grade II). The average annual concentration of PM2.5 from global satellite observations is used in our empirical research as a robustness check.

*URI_k,t_* is the urban-rural inequality of province *k* in year *t*, which are provincial indicators representing the income inequality of city *i* in province *k*. As the urban-rural income inequality constitutes over 70% of the overall regional income inequality in China [64,65,66], it is justified to use the urban-rural income ratio as a substitute variable for income inequality.

The vector *X_i,t_* represents a range of control variables related to a city’s air quality, including per capita gross regional product (*PCGRP*) at a constant price for the year 2014, the density of the population (*DP*), industry structure measured by the share of the secondary industry in the gross regional product (Secondary Industries Proportion, *SIP*), unit area coal consumption (provincial indicator, Coal Consumption, *CC*), greening rate (*GR*) of the urban built-up area, and level of financial development (*FD*) measured by per capita loans from financial institutions. Among them, *PCGRP* and *DP* represent social development, and other variables control the source of pollution.

### 3.3. Sample and Data

Air quality data of 113 key environmental protection cities from the National Environmental Air Quality Monitoring Network published in the China Statistical Yearbook are used to measure the dependent variables of urban air pollution. Because of widespread concern about smog in China, the Chinese government issued the new Environmental Air Quality Standard (GB3095-2012) and the Technical Specifications for Environmental Air Quality Assessment (Provisional) (HJ663-2013), which include seven indicators in the statistical coverage: SO_2_, NO_2_, PM10, CO, O_3_, PM2.5, and GAQD. These are the most authoritative annual continuous observation data on China’s air quality, covering major air pollutants. The summary statistics of air quality indicators of 113 key environmental protection cities in China are shown in Table 1. Environmental monitoring reports that Haikou is the city with the best air quality in China, while Baoding and Zibo are the cities with the most serious air pollution in recent years. Table 1 also report the unit root test results to address the concern of integrated of order 0 (I(0)). For the panel data with only 5 years, IPS test with t-bar statistic [67] and HT test with z-statistic [68] have been used. These test show that the empirical research of this paper will not be troubled by the unit root.

Compared with satellite observations widely used in smog research in China [69], the data from ground monitoring by the Ministry of Ecology and Environment of the People’s Republic of China in key environmental protection cities have an advantage in that the observations the of air pollutants can be obtained from ground monitoring, and information on changes in the structure of air pollutants can therefore be obtained. As a robustness test, this study also employs the average annual concentration of PM2.5 from global satellite observations. These data were obtained from the Air Quality Life Index, produced by the Energy Policy Institute at the University of Chicago (EPIC) (https://dev-aqli-epic.pantheonsite.io/the-index/?visitorCountryCode=CN&l=en, 15 November 2018).

The urban-rural inequality (*URI*) of the province is measured as the ratio of disposable income per capita of urban residents to per capita disposable income of rural residents. We use the regional urban-rural income ratio as a proxy for income inequality. Urban and rural income data are based on household surveys conducted by the National Statistical Bureau of China. Using the urban–rural income ratio as an inequality indicator is justified because the urban-rural income gap constitutes over 70% of the overall regional income in China [64,65,66]. Bourguignon and Morrisson [70] suggested that a major factor in country differences in income distribution is the labor productivity of non-agricultural sectors versus that of agriculture.

Data on the gross regional product, population, industry structure, green space, and loans were collected from China City Statistical Yearbooks (2015–2019). The provincial coal consumption data from 2014 to 2017 were collected from the *China Energy Statistical Yearbooks* (2015–2018). Coal consumption data for 2018 are not reported in statistical yearbooks; thus, it is calculated as the ratio of national coal consumption in 2018 and 2017, because the change in coal consumption is negligible over the two years (National coal consumption in 2017 is 3914.03 million tons, and in 2018, 3974.52 million tons). The correlation matrix and variance inflation factor (VIF) among these control variables is shown in Table 2. Since the correlation coefficient between variables is not particularly large, and all VIF are less than 10, this indicates that our regressions will not be affected by multicollinearity. The condition index for collinearity diagnostics among the variables reported the in Table 3 reinforces those results.

## 4. Results and Discussion

### 4.1. Income Inequality and Air Pollution

The individual fixed effect *u_i_* in the empirical model shown in Equation (1) represents factors affecting urban air quality that cannot be observed, such as atmospheric circulation and environmental capacity, which are constant in the time dimension. The Hausman test is used to confirm that the fixed effects model is better than the random effect model. The adjusted *R^2^* of the model shows a good fit, and the specific estimation results are shown in Table 4.

Despite differences in the sources and determinants of air pollution, the concentrations of most typical air pollutants are monitored, except for O_3_ (the O3 pollution in Chinese cities is mainly related to high temperature and photochemical smog generated by motor vehicle exhaust, which is quite different from other typical air pollutants) (Table 4), including SO_2_, PM10, CO, and PM2.5, are significantly positively associated with the urban-rural income inequality variable (*URI_i,t_*). The empirical results validate the hypothesis that the widening of income inequality increases the severity of air pollution and that income distribution deteriorates urban air quality, further worsening the negative effect of widening income inequality on social benefits.

Our results also show the impact of per capita GDP (ln*PCGRP_i,t_*) and urban population density (ln*DP_i,t_*) on air pollution in Chinese cities. Most of the air quality indicators have a significant and positive association with per capita GDP, including SO_2_, NO_2_, PM10, CO, and PM2.5, from ground monitoring and satellite observations. GAQD, the reverse indicator of air pollution, has a significant and negative association with per capita GDP. Thus, economic growth will increase the severity of China’s environmental pollution. The coefficient estimations of urban population density were significant in the regressions of PM10 and ground monitoring of PM2.5. Thus, there is a positive correlation between smog and urban population density even when controlling for other factors such as income inequality and per capita GDP.

The variables selected from air pollution sources show strong explanatory power with respect to urban air quality in the empirical model. Cities with a large share of secondary industry and those which use coal extensively experience serious air pollution, and financial development can reduce air pollution. However, public facilities investment, which uses the urban greening rate as a proxy variable, shows no effect on air pollution.

Hence, two empirical results are as follows:

**Result 1:** Income inequality aggravates air pollution measured by annual average PM2.5 concentration, from ground monitoring and satellite observations.

**Result 2:** Income inequality is positively correlated with annual average SO_2_ concentration, annual average NO_2_ concentration, annual average PM10 concentration, and the 95th percentile of daily average CO concentration. The empirical evidence further supports that besides PM2.5, income inequality is positively correlated with other air pollution indicators.

Therefore, hypothesis H2 is supported based on regression results of most of the air pollutants investigated**.** These results are similar to some previous studies, e.g., Zhang and Zhao [21] and Hao et al. [4]. These studies have demonstrated that inequality is associated with higher CO_2_ emission, and we extend it to some other typical pollutants. Contrarily, our results are inconsistent with those of Liu, et al. [38] and Yang et al. [37], who argue that wider inequality is associated with a lower pollution level.

### 4.2. Endogeneity Concerns

It appears that air pollution can aggravate income inequality [39]. Therefore, we have to examine whether the inclusion of income inequality in the model may suffer from the endogeneity problem. In other words, the causality between air pollution and income inequality could be bidirectional.

The TSLS allows us to address the endogeneity problem. The precision of TSLS estimation lies in the appropriateness of instrumental variables.

We use urbanization rate as the IV of *URI_k,t_*, where the rate of urbanization is the urban population/rural population ratio. The explained variables of our model involve air pollution in urban areas, which has no correlation with the urbanization rate in theory. The urban-rural inequality is an important factor driving the migration between urban and rural areas. There is a close relationship between urbanization rate and urban-rural income inequality, so the rate of urbanization can be used as an exogenous IV of urban-rural income inequality. Table 5 reports the results of the TSLS regression, and the under identification test, weak identification test and overidentification test show that the IV selected in our empirical study is valid. Our empirical results are robust when using instrumental variables and TSLS estimation.

### 4.3. Testing Environmental Kuznets Curves with Urban-Rural Inequality

**Figure 1:**
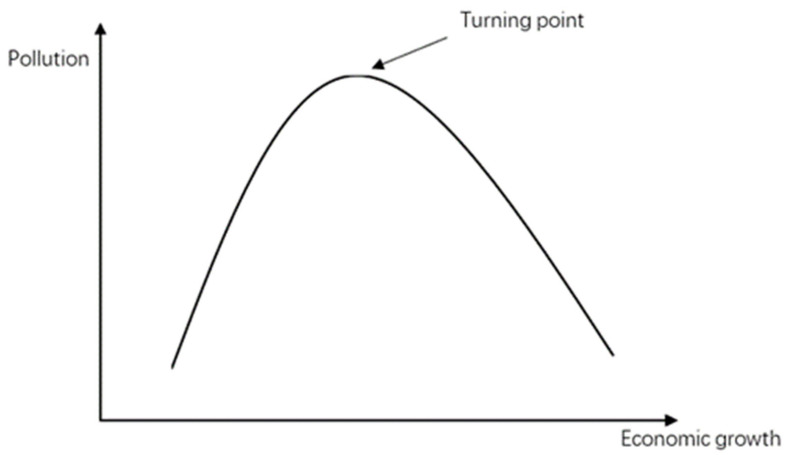
Environmental Kuznets curve (EKC).

According to the EKC hypothesis, there is an inverted U-shaped relationship between economic growth and environmental pollution, that is, environmental quality has a dynamic trend of deterioration first and then improvement with economic growth [10,11]. The EKC hypothesis comes from the observation of economic data. Grossman and Krueger [11] found no evidence that environmental quality deteriorates steadily with economic growth. Rather, for most indicators, economic growth brings an initial phase of deterioration followed by a subsequent phase of improvement. The EKC is exhibited in Figure 1.

The relationship between economic growth and the environment is likely to be non-linear. When testing the EKC hypothesis, the commonly used reduced-form is a quadratic function. See Equation (2).

(2) $$\begin{array}{cc} pollution_{i,t}= & f(PCGDP)\\ = & \beta_{0}+\beta_{2}\ln PCGRP_{i,t}+\beta_{3}(\ln PCGRP_{i,t}{)}^{2}+\varepsilon_{i,t} \end{array}$$

where, *pollution* is the environmental pollution variables. Once *β*_2_ > 0, *β*_3_ < 0 is observed in Equation (3), the EKC hypothesis is established, that is, environmental quality deteriorates first and then improves with economic growth. The turning point of EKC can be calculated by the value of *β*_2_ and *β*_3_, where the environment quality changes from deterioration to improvement.

To explore whether the EKC hypothesis is true for urban air pollution in China, we estimate the following econometric model that considers urban-rural inequality.

(3) $$AIR_{i,t}=\beta_{0}+\beta_{1}URI_{k,t}+\beta_{2}\ln PCGRP_{i,t}+\beta_{3}(\ln PCGRP_{i,t}{)}^{2}+\mu_{i}+\varepsilon_{i,t}$$

Estimation results of PCGRP and urban air pollution are shown in Table 6. Table 6 reports the regression result of SO_2_ and PM2.5, the two major air pollutants in China.

The regression results in Table 6 show that consistent with the main conclusions of this paper, the larger the urban–rural inequality, the more serious the air pollution from SO_2_, PM2.5 from ground monitoring and space observation, with the assumption of the same PCGRP (per capita gross regional product) and other control variables.

In addition, we observe that the EKC hypothesis is established in Table 6 because the statistical air pollutant indicators show that the air quality of Chinese cities deteriorates first and then improves with the economic growth. The regressions without control variables indicate that if the PCGRP exceeds RMB (Chinese currency) 45,214, the annual average concentration of PM2.5 from ground monitoring will decrease. The PCGRP of most Chinese cities has exceeded this standard, and they are in the stage of environmental quality improvement. The regressions with control variables show the turning point of PM2.5 from ground monitoring is RMB 159,072, that is, some cities in the developed areas have crossed the turning point.

Air pollution and the increase of an income gap are by-products of China’s economic growth and major concerns of the public. The emission characteristics of China’s provinces and cities are closely related to their different economic development and income levels, so the formulation and implementation of relevant policies should be proceeded with caution [71]. Fortunately, based on the results of this study, the policy ideas around solving these issues is not contradictory. There is a significant positive causal relationship between income inequality and air pollution, which means that reducing income inequality should effectively solve air pollution. It may also imply that there is no need to curb economic growth excessively for the sake of environmental governance. Sustainable economic development eventually narrows the income gap, and hence leads to the improvement of the environment. The regression results of other air pollutants based on the EKC hypothesis are shown in Table 7. Only PM10 shows similar results as SO_2_ and PM2.5, in which the air quality of Chinese cities improves first and then deteriorates with the economic growth. Therefore, hypothesis H1 is supported only for some of the pollutants.

## 5. Conclusions and Policy Implications

Overall, this paper has presented a triangulation of evidence on the impact of inequality on the environment. More specifically, income inequality aggravates air pollution measured by annual average PM2.5 concentration, both from ground monitoring and satellite observations. Income inequality is also positively correlated with other typical air pollutants (SO_2_, NO_2,_ PM10, CO). The results also show no evidence of the impact of per capita income on pollution. Our game-theoretic model and empirical results concur that reducing inequality improves environmental protection. Our empirical results are consistent with those of Zhang and Zhao [21] and Hao et al. [4], who have demonstrated that inequality is associated with higher CO_2_ pollution, but inconsistent with those of Liu et al. [38] and Yang et al. [37], who have demonstrated that inequality is associated with the lower levels of the broad-based environmental protection indices that they used.

We suspect that a reason our results are consistent with Zhang and Zhao [21] and Hao et al. [4] is that CO_2_ emissions are associated with the burning of fossil fuels, which is associated with SO_2_ and other air pollutants examined in this study. Unlike other pollutants, CO_2_ does not worsen air quality; thus, it is less likely to be of concern to China’s citizens who confront the reality of poor air quality every day. Future research should differentiate the impact of inequality between CO_2_ and other air pollutants. Regarding the inconsistency of our results with Liu et al. [38] and Yang et al. [37], further research should be conducted to compare the impact of using different units of analysis such as cities, provinces, and national-level data. In general, the main contribution of this study is that we presented evidence on the positive impact of income disparity on air pollution, which suggests that policies to improve the unequal income distribution may also have a beneficial environmental effect.

A limitation of our study is that it does not examine whether the results can be explained by our theoretical model or if other models provide a better explanation. One means to test Boyce’s [56] theory is to examine if the recent anti-corruption campaign in China led to a decrease in air pollution because the anti-corruption campaign was intended to weaken the power of corrupt government officials who became wealthy from accepting bribes from the rich and powerful. To test our model better, more research should be conducted on local government responsiveness when income in a city or region become more equal. Based on the results of this study and limitations mentioned above, follow-up investigation may focus on specific production and management activities from a more micro perspective and differentiate the different air pollutants. We will also focus on the relation between narrowing the income gap and achieving the goal of “emission peak and carbon neutrality”. Besides, how the trajectory of an environmental indicator in the EKC and the other trajectory of income inequality in the Kuznets Curve converge is also the topic we hope to work on in the future.

Several policy implications can be gleaned from our results. First, we find a strong impact of inequality on air pollution but no impact of per capita income on pollution. This finding suggests that it is not economic growth that affects pollution but the distribution of the growth. One effective measure to improve air quality may be to impose higher taxes on air polluters while allocating a portion of the revenue to lower-income individuals. The control variable that had the biggest impact on air pollution is the proportion of the secondary industry. This result suggests that the government should encourage economic development in industries other than the secondary industry. Another policy recommendation would be to invest in green technologies as a secondary industry which could improve energy efficiency and reduce emissions.

## Figures and Tables

**Table 1:** Summary Statistics of Air Quality Indicators of 113 Key Cities in China (2014–2018).

Air Quality Indicators	Obs	Mean	Media	SD	Min	Max	Unit Root Test	
							IPS t-Bar	HT z-Statistic
Annual average SO_2_ concentration (μg/m^3^)	565	25.5	21.0	16.6	5 (Haikou, 2015, 2018)	123 (Zibo, 2014)	−1.681	1.408
Annual average NO_2_ concentration (μg/m^3^)	565	37.3	37.0	10.3	12 (Haikou, 2017)	67 (Zibo, 2014)	−2.201 ***	−2.252 **
Average annual PM10 concentration (μg/m^3^)	565	90.9	86.0	30.5	35 (Haikou, 2016)	224 (Baoding, 2014)	−2.002 ***	−4.069 ***
95th percentile of average daily CO concentration (μg/m^3^)	565	1.99	1.7	0.85	0.8 (Haikou, 2017, 2018; Xiamen, 2017; Quanzhou, 2018)	5.8 (Baoding, 2015)	−1.629	-8.863 **
90th percentile of the daily maximum 8-h average O_3_ concentration (μg/m^3^)	565	149.8	149.0	27.2	69 (Hefei, 2014)	218 (Baoding, 2017)	−2.281 ***	−4.512 ***
Annual average PM2.5 concentration (μg/m^3^)	565	52.0	52.0	17.8	18 (Haikou, 2018)	129 (Baoding, 2014)	−2.117 ***	−3.793 ***
Number of days with air quality reaching or exceeding grade II	565	258	260	61.3	79 (Baoding, 2014)	366 (Panzhihua, 2016)	−2.332 ***	−7.844 ***

Note: Obs indicates the number of observations. SD indicates standard deviation. Min indicates minimum value. Max indicates maximum value. IPS indicates Im, Pesaran and Shin (2003). HT indicates Harris and Tzavalis (1999). Superscripts, **, and *** indicate significant levels of confidence at 5%, and 1%, respectively.

**Table 2:** Correlation matrix and variance inflation factor (VIF) among the control variables.

Variables	*URI_k,t_*	ln*PCGRP_i,t_*	ln*DP_i,t_*	*SIP_i,t_*	Ln*CC_k,t_*	*GR_i,t_*	ln*FD_i,t_*
*URI_k,t_*	1						
ln*PCGRP_i,t_*	−0.124	1					
ln*DP_i,t_*	−0.276	0.137	1				
*SIP_i,t_*	0.107	0.148	−0.187	1			
ln*CC_k,t_*	−0.464	0.067	0.303	−0.092	1		
*GR_i,t_*	−0.094	0.208	0.113	−0.049	0.162	1	
ln*FD_i,t_*	−0.123	0.655	0.359	−0.327	0.037	0.165	1
VIF	1.325	2.516	1.334	1.547	1.384	1.076	2.930

Note: *URI* indicates the urban-rural inequality. *PCGRP* indicates per capita gross regional product. *DP* indicates the density of the population. *SIP* indicates the secondary industries proportion. *CC* indicates coal consumption. *GR* indicates the greening rate of the urban built-up area. *FD* indicates per capita loans from financial institutions.

**Table 3:** Collinearity diagnostics among the control variables.

Dimension	Eigenvalue	Condition Index	Variance Proportions							
			Constant	*URI_k,t_*	ln*PCGRP_i,t_*	ln*DP_i,t_*	*SIP_i,t_*	ln*CC_k,t_*	*GR_i,t_*	ln*FD_i,t_*
1	7.893	1.000	0.00	0.00	0.00	0.00	0.00	0.00	0.00	0.00
2	0.053	12.187	0.00	0.00	0.00	0.01	0.52	0.02	0.01	0.00
3	0.025	17.827	0.00	0.19	0.00	0.01	0.08	0.26	0.00	0.00
4	0.012	25.830	0.00	0.02	0.00	0.16	0.00	0.01	0.82	0.00
5	0.010	27.755	0.00	0.20	0.00	0.38	0.03	0.51	0.05	0.00
6	0.005	38.380	0.01	0.33	0.02	0.38	0.00	0.05	0.12	0.06
7	0.001	86.937	0.66	0.22	0.00	0.00	0.12	0.15	0.01	0.33
8	0.000	137.694	0.32	0.04	0.97	0.07	0.24	0.00	0.01	0.60

Note: *URI* indicates the urban-rural inequality. *PCGRP* indicates per capita gross regional product. *DP* indicates the density of the population. *SIP* indicates the secondary industries proportion. *CC* indicates coal consumption. *GR* indicates the greening rate of the urban built-up area. *FD* indicates per capita loans from financial institutions.

**Table 4:** Regressions analysis for air pollution and income inequality.

Variables		SO_2_	NO_2_	PM10	CO	O_3_	PM2.5		GAQD
							Ground Monitoring	Satellite Observations	
Income Inequality	Urban–Rural Inequality *URI_k,t_*	65.885 *** (14.431)	2.906 (6.128)	52.685 *** (19.252)	1.936 *** (0.553)	−61.216 ** (26.815)	47.220 *** (12.195)	68.540 *** (9.718)	−61.609 (41.796)
Social development	Per capita gross regional product ln*PCGRP_i,t_*	10.560 ** (4.686)	3.290 * (1.990)	16.755 *** (6.252)	0.482 *** (0.179)	−2.992 (8.708)	11.884 *** (3.960)	6.066 * (3.156)	−37.253 *** (13.573)
	Population density ln*DP_i,t_*	1.585 (2.096)	−0.493 (0.890)	5.506 ** (2.797)	0.032 (0.080)	2.195 (3.895)	4.332 ** (1.771)	0.587 (1.412)	0.998 (6.071)
Source of pollution	Secondary industries proportion *SIP_i,t_*	0.871 *** (0.142)	0.155 *** (0.060)	1.461 *** (0.190)	0.025 *** (0.005)	−1.600 *** (0.264)	0.770 *** (0.120)	0.494 *** (0.096)	−2.094 *** (0.412)
	Coal consumption ln*CC_i,t_*	−3.509 (5.472)	5.218 ** (2.324)	13.351 * (7.301)	0.389 * (0.210)	15.615 (10.169)	15.820 *** (4.625)	4.219 (3.685)	−36.993 ** (15.850)
	Greening rate *GR_i,t_*	0.185 (0.130)	0.093 * (0.055)	0.293 * (0.174)	0.004 (0.005)	0.067 (0.242)	0.143 (0.110)	-0.093 (0.088)	−0.663 ** (0.378)
	Financial development ln*FD_i,t_*	−16.192 *** (3.495)	−2.081 (1.484)	−27.751 *** (4.663)	−0.747 *** (0.134)	25.445 *** (6.494)	−18.755 *** (2.954)	−14.110 *** (2.353)	27.366 *** (10.122)
Intercept term		−108.324 * (59.421)	−25.602 (25.233)	−116.844 (79.275)	−3.819 * (2.276)	−3.984 (110.416)	−160.914 *** (50.217)	−87.387 ** (40.015)	881.058 *** (172.104)
Urban fixed effect		Yes	Yes	Yes	Yes	Yes	Yes	Yes	Yes
Sample size		535	535	535	535	535	535	535	535
Adjusted *R*^2^		0.716	0.866	0.854	0.842	0.642	0.823	0.846	0.826

Note: Standard deviation is the estimated value in parentheses. Superscripts *, **, and *** indicate significant levels of confidence at 10%, 5%, and 1%, respectively. SO_2_ indicates the annual average sulfur dioxide concentration. NO_2_ indicates annual average nitrogen dioxide concentration. PM10 indicates the annual average inhalable particulate matter concentration. CO indicates the 95th percentile of daily average carbon monoxide concentration. PM2.5 indicates the annual average concentration of fine particulate matter. GAQD indicates the number of days with air quality reaching or exceeding grade II.

**Table 5:** TSLS regressions analysis for air pollution and income inequality.

Variables		SO_2_	NO_2_	PM10	CO	O_3_	PM2.5		GAQD
							Ground Monitoring	Satellite Observations	
Income inequality	Urban-rural inequality *URI_k,t_*	30.127 *** (8.460)	13.042 ** (5.181)	97.159 *** (19.136)	1.784 *** (0.449)	−22.270 * (12.854)	45.952 *** (10.883)	36.448 *** (9.540)	−157.248 *** (36.858)
Social development	Per capita gross regional product ln*PCGRP_i,t_*	−10.413 *** (2.443)	−3.288 ** (1.496)	−17.598 *** (5.527)	−0.581 *** (0.130)	3.825 (3.713)	−8.072 *** (3.143)	−0.337 (2.756)	16.526 (10.646)
	Population density ln*DP_i,t_*	0.215 (1.059)	3.471 *** (0.648)	10.803 *** (2.395)	0.126 ** (0.056)	1.842 (1.609)	9.262 *** (1.362)	10.334 *** (1.194)	−23.313 *** (4.613)
Source of pollution	Secondary industries proportion *SIP_i,t_*	0.256 *** (0.077)	0.039 (0.047)	0.389 ** (0.175)	−0.001 (0.004)	−0.078 (0.118)	0.199 ** (0.100)	0.066 (0.087)	-0.368 (0.337)
	Coal consumption ln*CC_i,t_*	9.658 *** (1.285)	4.942 *** (0.787)	21.420 *** (2.908)	0.360 *** (0.068)	10.712 *** (1.953)	10.538 *** (1.654)	9.410 *** (1.450)	−44.104 *** (5.601)
	Greening rate *GR_i,t_*	0.035 (0.142)	−0.142 * (0.087)	−0.411 (0.320)	0.003 (0.008)	0.098 (0.215)	−0.129 (0.182)	−0.104 (0.160)	0.524 (0.617)
	Financial development ln*FD_i,t_*	1.562 (1.692)	5.020 *** (1.036)	0.348 (3.827)	−0.009 (0.090)	−0.732 (2.571)	−3.437 (2.177)	−5.672 *** (1.908)	6.663 (7.372)
Intercept term		−31.346 (40.304)	−69.704 *** (24.682)	−180.246 ** 91.169)	0.788 (2.139)	86.987 (61.241)	−71.011 (51.849)	−113.085 ** (45.452)	844.158 *** (175.601)
Urban fixed effect		Yes	Yes	Yes	Yes	Yes	Yes	Yes	Yes
Sample size		535	535	535	535	535	535	535	535
*R* ^2^		0.747	0.941	0.871	0.861	0.977	0.877	0.856	0.935
IV		Urbanization rate							
Under Identification Test (*p*-value)		48.434 *** (0.000)							
Weak Identification Test		52.459 ***							
Overidentification Test		0.000							

Note: Standard deviation is the estimated value in parentheses. Superscripts *, **, and *** indicate significant levels of confidence at 10%, 5%, and 1%, respectively. SO_2_ indicates the annual average sulfur dioxide concentration. NO_2_ indicates annual average nitrogen dioxide concentration. PM10 indicates the annual average inhalable particulate matter concentration. CO indicates the 95th percentile of daily average carbon monoxide concentration. PM2.5 indicates the annual average concentration of fine particulate matter. GAQD indicates the number of days with air quality reaching or exceeding grade II.

**Table 6:** Regression analysis for EKC hypothesis: SO_2_ and PM2.5.

Variables	SO_2_		PM2.5			
			Ground Monitoring		Satellite Observations	
Urban-rural inequality *URI_k,t_*	125.708 *** (12.965)	67.327 *** (14.401)	119.465 *** (11.569)	48.910 *** (12.120)	111.960 *** (8.830)	69.787 *** (9.669)
Per capita gross regional product ln*PCGRP_i,t_*	184.394 *** (66.491)	134.438 ** (83.981)	224.738 *** (59.334)	157.044 *** (53.699)	150.678 *** (45.284)	113.121 *** (42.844)
Squared term of Per capita gross regional product ln*PCGRP_i,t_*^2^	−8.646 *** (2.985)	−5.596 * (2.974)	−10.483 *** (2.664)	−6.556 *** (2.419)	−7.234 *** (2.033)	−4.835 ** (1.930)
Population density ln*DP_i,t_*	——	0.980 (2.112)	——	3.623 ** (1.777)	——	0.064 (1.418)
Secondary industries proportion *SIP_i,t_*	——	0.864 *** (0.142)	——	0.762 *** (0.119)	——	0.488 ** (0.095)
Coal consumption ln*CC_i,t_*	——	−3.578 (5.454)	——	15.738 *** (4.590)	——	4.159 (3.662)
Greening rate *GR_i,t_*	——	0.197 (0.130)	——	0.156 (0.109)	——	-0.083 (0.087)
Financial development ln*FD_i,t_*	——	−15.610 *** (3.496)	——	−18.074 *** (2.942)	——	−13.608 *** (2.347)
Intercept term	−1273.248 *** (371.675)	−798.854 ** (359.566)	−1450.867 *** (331.667)	−969.884 *** (302.593)	−1020.639 *** (253.131)	−683.999 *** (241.421)
Urban fixed effect	YES	YES	YES	YES	YES	YES
Sample size	565	535	565	535	565	535
Adjusted *R*^2^	0.679	0.718	0.777	0.826	0.824	0.848
EKC	YES	YES	YES	YES	YES	YES
Turning Point of EKC	42,768 RMB	164,714 RMB	45,214 RMB	159,072 RMB	33,342 RMB	120,347 RMB

Note: Standard deviation is the estimated value in parentheses. Superscripts *, **, and *** indicate significant levels of confidence at 10%, 5%, and 1%, respectively. SO_2_ indicates the annual average sulfur dioxide concentration. PM2.5 indicates the annual average concentration of fine particulate matter.

**Table 7:** Regression analysis for EKC hypothesis: other air pollutants.

Variables	NO_2_	PM10	CO	O_3_	GAQD
Urban–rural inequality *URI_k,t_*	2.639 (6.138)	56.374 *** (18.977)	1.966 *** (0.553)	−62.347 ** (26.861)	−64.810 (41.790)
Per capita gross regional product ln*PCGRP_i,t_*	−19.659 (27.196)	333.577 *** (84.085)	3.000 (2.452)	−100.112 (119.016)	−312.163 * (185.165)
Squared term of Per capita gross regional product ln*PCGRP_i,t_*^2^	1.036 (1.225)	−14.307 *** (3.787)	−0.114 (0.110)	4.386 (5.360)	12.415 (8.340)
Population density ln*DP_i,t_*	−0.381 (0.900)	3.959 (2.783)	0.019 (0.081)	2.670 (3.940)	2.340 (6.129)
Secondary industries proportion *SIP_i,t_*	0.157 *** (0.060)	1.442 *** (0.187)	0.024 *** (0.005)	−1.594 *** (0.264)	−2.078 *** (0.411)
Coal consumption ln*CC_i,t_*	5.231 ** (2.325)	13.173 * (7.187)	0.387 * (0.210)	15.670 (10.173)	−36.838 ** (15.827)
Greening rate *GR_i,t_*	0.091 (0.055)	0.322 * (0.171)	0.004 (0.005)	0.058 (0.243)	−0.689 * (0.377)
Financial development ln*FD_i,t_*	−2.188 (1.490)	−26.265 *** (4.607)	−0.735 *** (0.134)	24.990 *** (6.521)	26.076 *** (10.145)
Intercept term	102.289 (153.250)	−1882.415 *** (473.816)	−17.854 (13.817)	537.260 (670.651)	2413.114 ** (1043.396)
Urban fixed effect	YES	YES	YES	YES	YES
Sample size	535	535	535	535	535
Adjusted *R*^2^	0.866	0.858	0.842	0.641	0.827
EKC	YES	YES	YES	YES	YES
Turning Point of EKC	——	115,548 RMB	——	——	——

Note: Standard deviation is the estimated value in parentheses. Superscripts *, **, and *** indicate significant levels of confidence at 10%, 5%, and 1%, respectively.

## Data Availability

The data presented in this study are available on request from the corresponding author.
